# The applicability of magnetic resonance imaging classification system (MRICS) for cerebral palsy and its association with perinatal factors and related disabilities in a Croatian population-based sample

**DOI:** 10.3325/cmj.2021.62.367

**Published:** 2021-08

**Authors:** Sanja Lovrić Kojundžić, Danijela Budimir Mršić, Ivana Jelovina, Benjamin Benzon, Maja Tomasović

**Affiliations:** 1 University Hospital of Split, Department of Health Studies, University of Split School of Medicine, Split, Croatia; 2 University Hospital Split, Split, Croatia; 3 Department of Anatomy, Histology and Embryology and Neuroscience, University of Split School of Medicine, Split, Croatia

## Abstract

**Aim:**

To investigate the association of cerebral palsy motor disorders, perinatal factors, and related disabilities with brain magnetic resonance imaging classification score (MRICS)-based groups in a population-based sample.

**Methods:**

The study enrolled children with cerebral palsy born from 2003 to 2015 treated at Split University Hospital who underwent brain MRI scanning. Perinatal data (plurality, birth weight, gestational age, and Apgar score) were collected from hospital records. Motor disorders of cerebral palsy (gross and fine motor function) and the related disabilities (intellectual status, speech and eating ability, epilepsy, vision and hearing status) were evaluated with neurological status assessment. Neuroimaging findings were presented as MRICS-based groups.

**Results:**

Of 115 enrolled children, an abnormal finding on brain MRI was confirmed in 95%, including white matter injury (66%), maldevelopments (13.9%), gray matter injury (9.6%), and miscellaneous findings (6.1%). Gross and fine motor function were not significantly associated with MRICS-based group. All related disabilities and perinatal factors, except Apgar score, were significantly associated with MRICS-based group.

**Conclusion:**

Brain MRICS-based groups were associated with perinatal risk factors and related disabilities of cerebral palsy, but not with common motor disorders. MRI classification score is a reliable diagnostic tool, which strongly correlates with perinatal factors and related disabilities of cerebral palsy.

Cerebral palsy (CP) represents a group of conditions characterized by permanent and non-progressive motor disorders, which present as difficulties in motion, balance, and posture. CP has a range of etiologic pathways and associated disabilities. CP patients commonly suffer from intellectual, speech, vision, or hearing impairment or epilepsy, which makes the patient care complex and patient-specific. CP incidence is 1-3/1000, with a slightly decreasing trend in recent years (1). Several investigations suggested a different incidence rate and distributions of CP types, associated with socioeconomic factors and improvement in perinatal and natal care (2).

Of four main CP types (spastic, athetoid, ataxic, and mixed), spastic is the most common, accounting for up to 80% of the cases (3,4). The spastic type is related to motor cortex damage, athetoid to basal ganglia damage, ataxic to cerebellum damage, and mixed to combination damage. The affected limb is associated with the location of a specific brain injury. Neuroimaging represents a standard diagnostic tool in CP. Brain magnetic resonance imaging (MRI) findings are abnormal in a high percent of CP patients (5,6). MRI can detect anatomic abnormalities and help better understand the etiology and pathogenesis of CP. Based on the investigations in the last decades, MRI interpretation significantly progressed with the introduction of an MRI classification score (MRICS) by Surveillance of Cerebral Palsy in Europe (SCPE) in 2017. The score is designed to unify the MRI interpretation by specialists dealing with CP patients (7). MRI findings were divided into five groups based on the chronological order of brain damage appearance (A. Maldevelopments; B. Predominant white matter injury; C. Predominant gray matter injury; D. Miscellaneous; E. Normal). Some groups were subdivided depending on the extent of the lesion. So far, the applicability of the proposed MRICS has not been widely investigated (8).

To our knowledge, the incidence, and clinical and MRI characteristics of CP in southern Croatia have not been investigated so far. In general, CP research involving other Croatian regions is lacking (9,10). MRICS applicability is yet to be proven. Therefore, this study aimed to associate CP motor disorders, perinatal factors, and related disabilities of CP with MRICS-based groups in order to assess score applicability in a population-based sample in southern Croatia.

## MATERIALS AND METHODS

### Study design and participants

This retrospective population-based study was approved by the Ethics Committee of Split University Hospital Center. All procedures conformed to the ethical standards of the 1964 Helsinki Declaration and its later amendments or comparable ethical standards. Given the retrospective, non-interventional research setting, the informed consent was waived.

The study enrolled children with CP treated at the Department of Pediatrics, Split University Hospital, who were born from January 1, 2003 to December 31, 2015. The Department is a referral center for children with disabilities for the southern Croatian region, Dalmatia. The Department's CP register follows the SCPE guidelines regarding the CP definition and inclusion and exclusion criteria (11). The study included children with confirmed CP at the age of 5 years who underwent at least one brain MRI. CP was classified into five subtypes: bilateral spastic, unilateral spastic/left or right, dyskinetic, and ataxic.

Demographic and perinatal data were collected from paper-based and electronic medical records (sex, plurality, birth weight, gestational age, and Apgar score measured at five minutes). Apgar score values were classified as Apgar score 0-3, Apgar score 4-6, and Apgar score 7-10.

Motor disorders of CP were evaluated with neurological status assessment by experienced neuropediatricians and included the following: gross motor function (five levels according to the Gross Motor Function Classification System [GMFCS] – level 1 and 2: ability to walk without assistive devices; level 3: ability to walk with an assistive device; level 4 and 5: inability to walk even with devices) (12) and fine motor function (five levels according to the Bimanual Fine Motor Function [BFMF] – level 1: almost no manipulation restriction to level 5: hard restriction) (13).

Related disabilities were also evaluated with neurological status assessment, performed at a median of 8 years (Q1-Q3 5.5-9.0), and included the following: intellectual status (normal [IQ≥70]; mild [IQ 50-70]; moderate [IQ 35-50]; severe intellectual disability [IQ<35]) assessed with the Wechsler Intelligence Scale for Children and clinical judgment; speech ability (Viking Speech Scale: normal 0, indistinct speech 1, obviously indistinct speech 2, severely indistinct speech 3, no speech 4); eating ability (independent, needs assistance, or partial/full tube feeding); vision and hearing status (normal, impaired, or severely impaired); and epilepsy (present or not present).

MRI scanning was performed with Siemens 1.5 T Magnetom Avanto (Erlangen, Germany). Standard imaging protocol in our Institution included the following sequences: T1-weighted image (T1WI), T2-weighted image (T2WI), fluid-attenuated inversion recovery (FLAIR), and diffusion-weighted imaging/apparent diffusion coefficient image (DWI/ADC) in axial planes, T2-weighted (T2WI) in the coronal plane, and sagittal T1-weighted (T1WI). Additional sequences were used whenever necessary (ie, coronal inversion recovery sequence for assessing maldevelopments and T2*-W gradient-echo sequence in the case of hemorrhage or vascular lesion, or time-of-flight sequence to detect the etiology of intraparenchymal hemorrhage). The protocol was adjusted for younger children (those under 2 years of age) using short-tau inversion recovery (STIR) sequence instead of T2-W. Most of the children were scanned during sedation and required general anesthesia. The median age at MRI scanning was 3 years (Q1-O3, 1.0-4.5). A small number of neonates (under 2 months) were scanned during natural sleep. Contrast medium (gadolinium-based, Omniscan, single-dose 0.1 mmol/kg) was applied wherever necessary. Imaging data were extracted from the imaging database. Neuroimaging findings were reevaluated by a neuroradiology subspecialist (SLK) and divided into five groups (A. Maldevelopments; B. Predominant white matter injury; C. Predominant gray matter injury; D. Miscellaneous; E. Normal) and several subgroups (ie, A1, B1, B2, B3, C1, C2, C3), which were based on MRICS (7).

### Statistical analysis

Data are presented as percentages and absolute numbers, and continuous variables as median and interquartile range. Differences in continuous variables were tested with the Kruskal-Wallis test or Dunn’s test. In order to test for trends in ordinal variables we used linear and quadratic test. Differences in proportions were tested with χ^2^ test. Measures of significance were *P* values and R^2^. *P* < 0.05 was considered to be statistically significant. *P* values were interpreted according to the ASA Statement on *P* values (14). Data analysis was performed with SPSS, version 14 (SPSS, Chicago, IL, USA).

## RESULTS

The study enrolled 115 children; 69 (60%) were male. There was no significant sex difference in CP type and MRICS groups (*P* = 0.974). The majority of children (98 or 85%) were single births and 17 (15%) were multiple births (twins or more) (*P* < 0.050, Table 1).

**Table 1:** Cerebral palsy types, and demographic and perinatal characteristics in relation to magnetic resonance imaging classification score (MRICS) subgroups. Values are counts (%)*

	A1	B1	B2	B3	C1	C2	C3	D	E	P^†^
**Total N**	16	49	6	21	1	2	8	7	5	
**CP subtypes**										<0.05
**spastic bilateral**	7 (43.8)	40 (81.6)	4 (66.7)	16 (76.2)	0 (0.0)	0 (0.0)	0 (0.0)	5 (71.4)	2 (40.0)	
**spastic unilateral left**	3 (18.8)	1 (2.0)	2 (33.3)	2 (9.5)	0 (0.0)	0 (0.0)	4 (50.0)	0 (0.0)	0 (0.0)	
**spastic unilateral right**	2 (12.5)	5 (10.2)	0 (0.0)	1 (4.8)	1 (100)	1 (50.0)	3 (37.5)	0 (0.0)	0 (0.0)	
**dyskinetic**	3 (18.8)	2 (4.1)	0 (0.0)	2 (9.5)	0 (0.0)	0 (0.0)	1 (12.5)	2 (28.6)	2 (40.0)	
**ataxic**	1 (6.3)	1 (2.0)	0 (0.0)	0 (0.0)	0 (0.0)	1 (50.0)	0 (0.0)	0 (0.0)	1 (20.0)	
**Sex**										0.9735
**male**	9 (56.3)	32 (65.3)	3 (50.0)	11 (52.4)	1 (100)	1 (50.0)	5 (62.5)	4 (57.1)	3 (60.0)	
**female**	7 (43.8)	17 (34.7)	3 (50.0)	10 (47.6)	0 (0.0)	1 (50.0)	3 (37.5)	3 (42.9)	2 (40.0)	
**Plurality**										<0.05
**singleton**	14 (87.5)	38 (77.6)	4 (66.7)	18 (85.7)	1 (100)	2 (100)	8 (100.0)	7 (100.0)	5 (100.0)	
**twins**	2 (12.5)	11 (22.4)	2 (33.3)	3 (14.3)	0 (0.0)	0 (0.0)	0.0	0 (0.0)	0 (0.0)	
**Gestational age (weeks)**										<0.0001
**median**	39.5	31	29	32	40	38	40	38	40	
**25% percentile**	38	28.5	27.5	29.5	40	36	39.25	30	36	
**75% percentile**	40	35	34.5	35	40	40	40.75	40	41	
**Body weight (g)**										0.0005
**median**	3000	1550	1400	1700	NA	2400	3300	3575	3050	
**25% percentile**	2733	1200	1075	1350	NA	2400	3300	2225	2000	
**75% percentile**	3700	2070	2515	2370	NA	2400	3800	4325	4100	
**Apgar score**										0.4158
**median**	3	2	1	2	0	1.5	0	2	2	
**25% percentile**	0	0	0	0	0	0	0	0	0	
**75% percentile**	3	3	3	3	0	3	0	3	3	

*Subgroups based on MRICS: A1 – disorders of cortical formation, B1 – periventricular leukomalacia (PVL), B2 – intraventricular hemorrhage (IVH), B3 – PLV and IVH, C1 – basal ganglia/thalamus lesion, C2 – watershed infarction, C3 – arterial infarct, D – atrophy or delayed myelination, E – normal.

†Kruskal-Wallis test (Omnibus).

**Figure 1:**
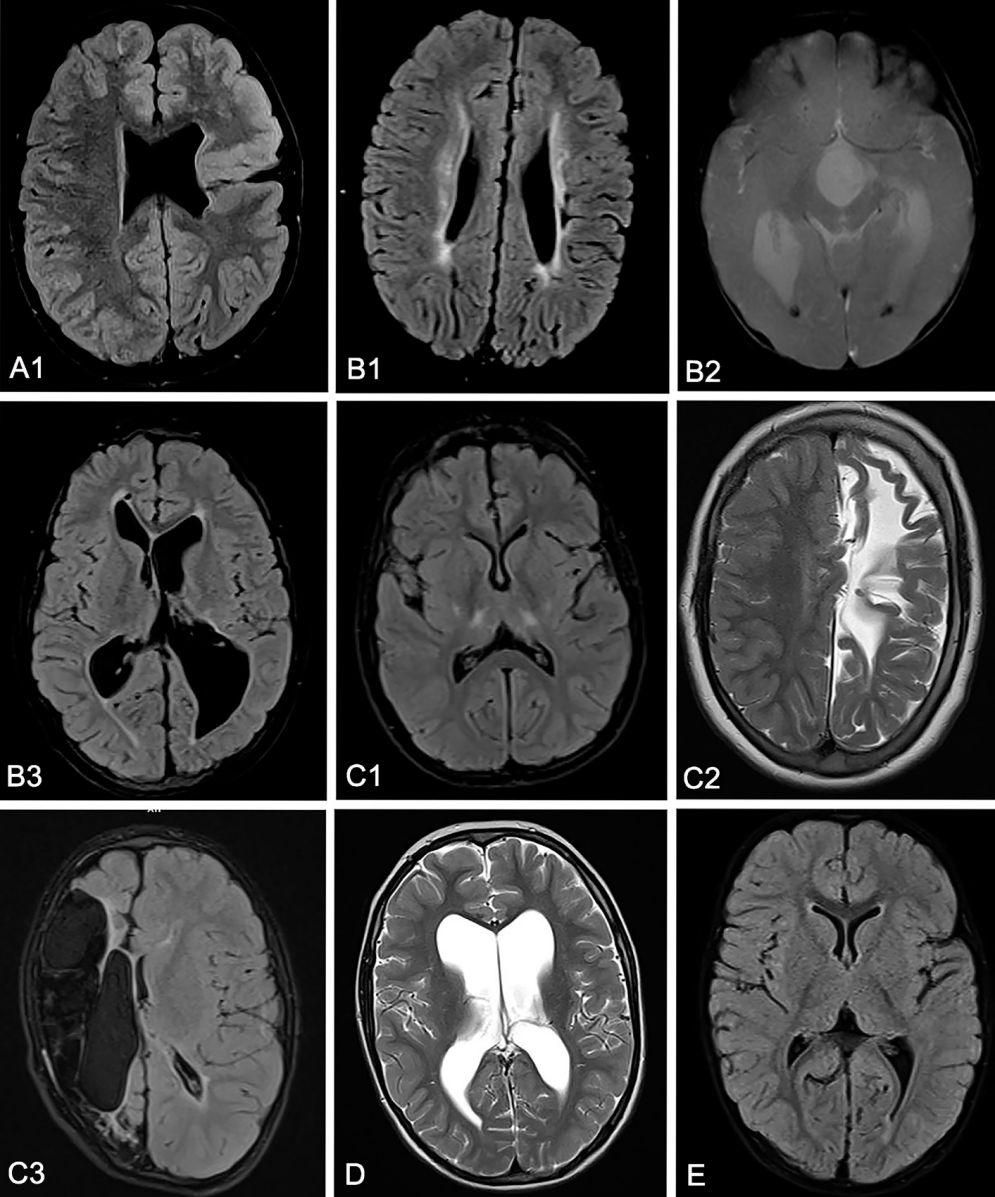
Examples of magnetic resonance imaging (MRI) findings of each MRI classification score-based group and subgroup (A-E): A1: axial fluid-attenuated inversion recovery (FLAIR) – open lip schizencephaly; B1: axial FLAIR – open periventricular leukomalacia (PVL) grade II; B2: axial T2* weighted image (WI) – sequelae of intraventricular hemorrhage; B3: axial FLAIR – a combination of PVL and IVH sequelae; C1: axial FLAIR – bilateral thalamic lesions; C2: axial T2 WI – parasagittal lesion; C3: axial T2 WI – middle cerebral artery infarction; D: axial T2 WI – ventriculomegaly; E: axial FLAIR – normal brain.

An abnormal finding on brain MRI was confirmed in 95% of children, including the presence of all five MRICS-based groups (Figure 1). White matter injury (group B) was a predominant pattern (66%), followed by maldevelopments (13.9%), gray matter injury (9.6%), miscellaneous (6.1%), and normal findings (4.4%) (Table 1). Maldevelopments constituted only A1 subgroup or disorders of cortical formation and included schizencephaly with polymicrogyria (N = 4), polymicrogyria (N = 3) schizencephaly (N = 3), cortical dysplasia (N = 3), heterotopia (N = 2), and lissencephaly (N = 1).

The most common CP subtype was spastic bilateral type (in 74 children, 60.1%), followed by spastic unilateral type (in 25 children, 21.7%), dyskinetic (in 12 children, 10.4%), and ataxic type (in 4 children, 3.5%) (*P* < 0.050). To assess possible neuroanatomical changes in specific CP type, brain MRI was performed.

The MRI findings of 74 children with spastic bilateral type showed the presence of every MRICS-based group, except gray matter injuries group. The most prevalent group was B1 (periventricular leukomalacia, PVL, in 81.6% of children), followed by miscellaneous group, ie, atrophy or delayed myelination (71.4% *P* < 0.050). This implies that spastic bilateral type was associated mainly with white matter injuries, and not with gray matter injuries. This was not the case for spastic unilateral type, as the MRI findings of 25 children with this type showed the presence of white matter injuries and gray matter injuries, followed by maldevelopments (*P* < 0.050). The MRI findings of 12 dyskinetic children and of 4 ataxic children showed almost equal prevalence of all MRICS-based groups without predominant associations.

Motor impairment of CP was not significantly associated with any of the MRICS-based groups. The median GMFCS was level 3 in the majority of the MRI groups (ranging from 2 to 5), indicating a partial ability to walk and an occasional need for assistance (or assistive device) in the majority of children. However, it was not significantly correlated with MRICS groups. A similar trend was observed with BFMF. The median level of BMFF was level 3, indicating a moderately affected fine motor function. Only group A1 had median-4 or severely reduced fine motor function. In addition, these observations were not significantly related to MRICS-based groups (*P* = 0.254).

Of perinatal factors, the lowest gestational age was significantly associated with white matter injuries group. The median gestational age was significantly lower in all white matter injury subgroups (in B1 – 31 weeks, in B2 – 29 weeks, and B3 – 32 weeks; all preterm births). The median gestational age in other MRICS groups (A, C, D, E) was from 38 to 40 weeks (term birth). Birth weight followed the same association pattern. The lowest birth weight was observed in children with white matter injuries and associated with their gestational age (*P* < 0.0001). Children in the B2 subgroup (intraventricular hemorrhage) had the lowest birth weight (median was 1400 g). Two other B subgroups had moderately low birth weight: the median of subgroup B1 was 1550 g and of subgroup B3 was 1700 g. Contrary to this, other MRICS groups (A, C, D, E) were associated with normal birth weight (*P* = 0.0005) (Table 1). The majority of children had Apgar values between 4 and 6, but Apgar score was not significantly associated with MRI findings (*P* = 0.416).

Related disabilities were significantly associated with MRICS-based groups. Normal intellectual ability was found in nearly half of the participants in the white matter injuries group, ranging from 42.6% in B3 to 66.7% in B2, followed by 40.1% in the normal MRI group. The highest rate of severe intellectual impairment was found in the miscellaneous injuries group (42.9%, *P* = 0.0002, Table 2). Impaired vision was present in all MRICS groups in a high percentage (>70%), mostly in the gray matter injuries group. Hearing ability was preserved, with a low prevalence of impairment among all MRICS groups (<20%, *P* < 0.001, Table 2). More than two-thirds of children with a cortical malformation (A1 group) and intraventricular hemorrhage (B2 group) were not able to eat without assistance, and the most independent eating was observed in children with gray matter injuries (subgroups C1 and C3, *P* = 0.03, Table 2). The highest proportion of epilepsy was found in the cortical malformations group (A1 subgroup, 75%) and in all three C subgroups (ie, gray matter injuries). Children with white matter injuries less frequently had epilepsy (*P* = 0.034, Table 2). Speech ability was differently affected, ranging from mild impairment in white matter injury group to more severe impairment in gray matter injuries group (median was 4 – no speech at all, *P* = 0.035, Table 2).

**Table 2:** Related disabilities of cerebral palsy in relation to MRI classification score (MRICS) subgroups. Values are counts (%)*

	A1	B1	B2	B3	C1	C2	C3	D	E	P value
Total N	16	49	6	21	1	2	8	7	5	
IQ										0.0002‡
normal	4 (25.0)	27 (55.1)‡	4 (66.7)	9 (42.9)	0 (0.0)	0 (0.0)	3 (37.5)	2 (28.6)	2 (40.09)	
mildly impaired	3 (18.8)	8 (16.3)‡	1 (16.7)	5 (23.8)	0 (0.0)	0 (0.0)	1 (12.5)	1 (14.3)	0 (0.0)	
moderately impaired	6 (37.5)	11 (22.4)‡	1 (16.7)	2 (9.5)	1 (100)	2 (100)	3 (37.5)	1 (14.3)	1 (20.0)	
severely impaired	3 (18.8)	3 (6.1)‡	0 (0.0)	5 (23.8)	0 (0.0)	0 (0.0)	1 (12.5)	3 (42.9)	2 (40.0)	
Vision										<0.001§
normal	5 (31.3)	15 (30.6)§	1 (16.7)	1 (4.8)§	0 (0.0)	0 (0.0)	1 (12.5)	2 (28.6)	1 (20.0)	
impaired	8 (50.0)	31 (63.3)§	4 (66.7)	15 (71.4)§	1 (100)	2 (100)	3 (37.5)	3 (42.9)	4 (80.0)	
severely impaired	3 (18.8)	3 (6.1)§	1 (16.7)	5 (23.8)§	0 (0.0)	0 (0.0)	4 (50)	2 (28.6)	0 (0.0)	
Hearing										<0.001‡
normal	14 (87.5)‡	48 (97.6)‡	6 (100)‡	19 (89.5)‡	0 (0.0)	1 (50)	8 (100)‡	4 (60.0)	4 (80.0)	
impaired	2 (12.5)‡	1 (2.4)‡	0 (0.0)‡	2 (10.5)‡	0 (0.0)	1 (50)	0 (0.0)‡	1 (20.0)	0 (0.0)	
severely impaired	0 (0.0)‡	0 (0.0)‡	0 (0.0)‡	0 (0.0)‡	1 (100)	0 (0.0)	0 (0.0)‡	1 (20.0)	1 (20.0)	
Feeding ability										0.03§‡
normal	5 (31.3)‡	25(50.0)§	2 (33.3)	11 (52.6)	1 (100)	0 (0.0)	5(57.1)	1 (16.7)‡	2 (40.0)	
partly need assistance	0 (0.0)‡	5 (9.5)§	0 (0.0)	0 (0.0)‡	0 (0.0)	0 (0.0)	0 (0.0)	0 (0.0)‡	0 (0.0)	
mainly need assistance	11 (68.8)‡	20 (40.5)§	4 (66.7)	10 (47.4)	0 (0.0)	2 (100)	3 (42.9)	6 (83.3)‡	3 (60.0)	
Epilepsy										0.0342
no	4 (25.0)	26 (53.1)	4 (66.7)	11 (52.4)	0 (0.0)	1 (50)	4 (50.0)	4 (57.1)	3 (60.0)	
yes	12 (75.0)	23 (46.9)	2 (33.3)	10 (47.6)	1 (100)	1 (50)	4 (50.0)	3 (42.9)	2 (40.0)	
Gross Motor Function Classification System										0.2191†
median	3	3	2.5	3	5	2	2	5	2	
25% percentile	1.25	2	1	2	N/A	2	1	3	1.5	
75% percentile	4.75	4	4.25	5	N/A	2	4	5	4	
Bimanual Fine Motor Function										0.2538†
median	4	2	2	3	5	3	3	3	2	
25% percentile	2	1	1	1	N/A	3	2	2	1	
75% percentile	4	3	3.25	4	N/A	3	4	4	4	
Speech scale										0.0358†
median	3	2	1.5	2	4	4	2	3	3	
25% percentile	1.25	1	1	1	4	4	2	1	1	
75% percentile	4	3	2	3	4	4	4	4	4	

*Subgroups based on MRICS: A1 – disorders of cortical formation, B1 – periventricular leukomalacia (PVL), B2 – intraventricular hemorrhage (IVH), B3 – PLV and IVH, C1 – basal ganglia/thalamus lesion, C2 – watershed infarction, C3 – arterial infarct, D – atrophy or delayed myelination, E – normal.

†Kruskal-Wallis test (omnibus).

‡test for linear trend.

§test for quadratic trend.

χ^2^ test.

## DISCUSSION

The results of this study in a population-based sample in southern Croatia showed the highest prevalence of bilateral spastic type (60.1%), followed by unilateral spastic type (21.7%). Dyskinetic and ataxic types occurred in a smaller percentage (10.4% and 3.5%, respectively). An abnormal finding on brain MRI was confirmed in 95% of children, including the presence of all five MRICS-based groups. White matter injury was the predominant pattern (66%), followed by maldevelopments (13.9%), gray matter injury (9.6%), miscellaneous (6.1%), and normal findings (4.4%). All the mentioned frequencies and distributions of brain tissue damage were similar to the results in the European population, which confirms white matter injuries as the most common pathogenetic pattern of CP (5,8,15). Gray matter injuries and maldevelopments were the next most prevalent common pathogenetic imaging patterns in other studies as well (5,8,15).

Motor disorders in CP according to GMFCS and BFMF did not correlate significantly with MRI imaging. However, the non-significant association in our research is not difficult to explain, as previous research found a similar inconsistent association of motor function and brain MRI (16).

White matter injuries are strongly associated with spastic types of CP (17). Prematurity and low birth weight were significant risk factors for white matter injuries. Contrary to this, term gestational age and normal birth weight were associated with gray matter injuries, maldevelopments, and normal MRI findings. These associations agree fully with the literature (8,15). Plurality seemed not to increase the chance for CP development, as a majority of children were single births (84.4%). Sex distribution showed negligible male predominance (60% male).

Children with CP suffer from multiple problems and potential disabilities due to developmental delays. This study showed intellectual status, vision, hearing, speech and feeding abilities, and epilepsy occurrence to be associated with MRICS-based groups. Published research of associated disabilities with a particular MRICS-based group is lacking.

This study showed that intellectual status was generally not impaired in children with white matter injuries. This group mostly consisted of preterm children born at a low gestational age. Further, moderately-to-severely impaired intellectual status was found in children with gray matter injuries, miscellaneous group, and normal group. These results are fully consistent with other studies (8,18,19). Current scientific opinion is that both white and gray matter play significant roles in the development of human intelligence. However, most authors agree that gray matter, including its total volume or some specific gray matter areas, is the main determinant of intellectual functions. White matter provides only inter-neural connection pathways, thus supporting and facilitating these functions (20). Preterm children with white matter injuries from our study had a spectrum of intellectual statuses, ranging from normal to severely diminished, a finding that warrants further research on the exact role of white matter in the development of human intelligence.

Vision impairment showed a high prevalence in all MRICS-based groups, while hearing ability was largely not impaired. The incidence of visual impairment within the CP population is high. Numerous studies found a positive correlation between vision and motor impairment in CP (9,21). The most frequent pathogenic mechanism was PVL, affecting white matter that surrounds the posterior horns (21). Except the optic radiation pathway, the human brain cortex is also responsible for visual function, including the occipital lobe and some parts of the temporal and parietal lobes. Brain areas whose injury determines the motor deficit resulting in CP are anatomically closely located to the network of brain areas responsible for visual function (21). MRI is very sensitive in the detection of disorders of white or gray matter, thus predicting visual impairment. However, the latest review showed an overall low level of evidence correlating different patterns of visual impairment in CP and MRI, which requires future investigations (22). Hearing ability was often unaffected in other studies as well, which found less than 20% (23) or 39% (24) of hearing loss prevalence in CP.

Feeding problems are very common in people with CP (occurring in about 50%), posing an increased risks of malnutrition and dehydration, aspiration pneumonia, and poor quality of life (25). A similar percentage of patients needing assistance while feeding was found in our sample, commonly in the maldevelopment and miscellaneous group.

Epilepsy is common in CP, occurring in up to 50% of patients (26). We found epilepsy most often in the maldevelopments (a disorder of cortical formation) group, which is partly consistent with previous research. Nagy et al (8) found somewhat greater epilepsy prevalence in general, and in other MRCIS-based groups as well. Speech, assessed with speech scale, was significantly impaired in all MRICS-based groups, ranging from a median of 1.5 (in white matter injury) to 4 (in gray matter), which was expected due to the motor impairments in CP, especially in gray matter injuries.

The major limitation of this study is a relatively small number of some CP types in our population-based sample, such as dyskinetic and ataxic types. The second important limitation is that we did not use the appreciated clinical classification systems for functional assessment of children with CP. Manual Ability Classification System, Eating and Drinking Ability Classification System, and Communication Function Classification System are regularly used classification tools for evaluating domain-specific function level in children with CP, but they were not used in this study to assess participants' domain-specific function level.

In conclusion, classification score-based brain MRI groups were associated with CP types. The most frequent was the association between white matter injury and spastic cerebral palsy type. However, no association was found between motor impairment of CP and MRICS-based groups. Perinatal factors played an important role in CP occurrence and were strongly associated with MRICS-based groups, except the Apgar score. All associated disabilities of CP were significantly associated with brain MRICS-based groups. These results suggest that MRI classification score is a reliable diagnostic tool, which correlates strongly with perinatal factors and associated disabilities of CP.
